# Digital Technologies To Enhance Social Connectedness in Long-Term Care Facilities During COVID-19: A Review

**DOI:** 10.1093/geroni/igaa057.3451

**Published:** 2020-12-16

**Authors:** Shannon Freeman, Aderonke Abgoji, Alanna Koopmans, Christopher Ross

**Affiliations:** University of Northern British Columbia, Prince George, British Columbia, Canada

## Abstract

A consequence of the strict visitor restrictions implemented by many Long-term Care Facilities (LTCFs), during the COVID-19 pandemic, was the exacerbation of loneliness and social isolation felt by older adult residents. While there had been a shift by some persons to utilize digital solutions to mitigate the effects of the imposed social isolation, many facilities did not have sufficient information regarding available solutions to implement institutional strategies to support social connectedness through digital solutions. To support our partners in evidence-based policy-making we conducted a scoping review to identify existing virtual technology solutions, apps, and platforms feasible to promote social connectedness among persons residing in a long-term care facility context during times of lockdown such as experienced during the COVID-19 pandemic. Initial identification of relevant literature involved a combination of keywords and subject headings searches within 5 databases (PubMed, CINAHL EBSCO, PsychINFO EBSCO, Embase OVIDSP, and Web of Science ISI). DistillerSR was used to screen, chart and summarize the data. There is growth in the availability of technologies focused on promoting health and well-being in later life for persons in long-term care facilities however a gap remains in widespread uptake. We will describe the breadth of technologies identified in this review and discuss how they vary in utility in smaller scale facilities common in rural areas. Of the technologies that can be used to mitigate the impacts of social isolation felt by long-term care residents, many “solutions” depend on stable highspeed internet, which remains a challenge in rural and northern areas.

